# Expanding lateral access spine surgery

**DOI:** 10.3171/2022.4.FOCVID2232

**Published:** 2022-07-01

**Authors:** Laura A. Snyder, Melissa Erickson, Justin S. Smith, Praveen V. Mummaneni

**Affiliations:** 1 Department of Neurosurgery, Barrow Neurological Institute, St. Joseph’s Hospital and Medical Center, Phoenix, Arizona;; 2 Department of Neurosurgery, Duke University Medical Center, Durham, North Carolina;; 3 Department of Neurosurgery, University of Virginia Medical Center, Charlottesville, Virginia; and; 4 Department of Neurosurgery, University of California, San Francisco, California

Lateral approaches to the spine have been increasingly recognized as a minimally invasive way to treat spinal pathology and improve patient outcomes. Even as the number of spine cases using the lateral approach increases internationally, many spine surgeons are reticent to adopt lateral approaches as they may have not had exposure to these techniques in their training. Furthermore, if this training did occur, it may have been limited to more straightforward degenerative lateral techniques at the midlumbar levels, and thus for these surgeons more advanced lateral techniques may remain daunting. In this issue of *Neurosurgical Focus: Video*, the editors hope to provide readers with tools, tips, and techniques to advance their lateral access spine surgery practice as well as treat pathologies via lateral techniques in which they may have not considered previously. From thoracic disc treatment via a lateral retropleural technique to a transthoracic endoscopic approach, and from treating infection to scoliosis to schwanomma, this issue highlights how the lateral access approach can be utilized effectively for difficult pathology. As the videos emphasize safety and nuances, whether in the thoracic space, at the thoracolumbar junction, or when transitioning to prone lateral, the editors hope that this issue will help readers further adopt advanced lateral access surgery confidently.

## Disclosures

Dr. Mummaneni is a consultant for DePuy Synthes, Stryker, and Globus; has received royalties from DePuy Synthes, Thieme Publishers, and Springer Publishers; has directly purchased stock in Spinicity/ISD; and has grants from AO Spine, NREF, and NIH. Dr. Erickson has received consultant fees from DePuy Synthes, Medtronic, and Globus; receives fellowship funding from Medtronic and NuVasive; and owns stock in Restor3D. Dr. Smith has received consultant fees from Zimmer Biomet, NuVasive, Cerapedics, SeaSpine, and Stryker; receives royalties from Zimmer Biomet, NuVasive, and Thieme; receives research funding from DePuy Synthes/ISSGF, NuVasive, and AO Spine; and owns stock in Alphatec and NuVasive. Dr. Snyder is a consultant for Globus, Medtronic, and NuVasive; and receives research funding from Biogen.

